# Hyaluronic Acid Inhibits Polycation Induced Cellular Responses

**DOI:** 10.1155/S0962935194000396

**Published:** 1994

**Authors:** A. lalenti, A. lanaro, G. Brignola, P. Marotta, M. Di Rosa

**Affiliations:** Department of Experimental Pharmacology University of Naples ‘Federico II’ Via Domenico Montesano Naples 49-80131 Italy

## Abstract

Positively charged macromolecules cause a variety of pathological events through their electrostatic interaction with anionic sites present on the membrane of target cells. In the present study we have investigated the effect of hyaluronic acid, a negatively charged molecule, on rat paw oedema induced by poly-L-lysine as well as on histamine release from rat mast cells and nitric oxide formation from rabbit aorta, both induced by this polycation. The results indicate that hyaluronic acid is able to suppress these poly-L-lysine induced effects with a mechanism which possibly depends on its negative charges which may balance the effects of positively charged polycations.


Mediators of Inflammation 3, 287–289 (1994)

### Hyaluronic acid inhibits polycation induced cellular responses

A. Ialenti,[CA] A. Ianaro, G. Brignola, P. Marotta and M. Di Rosa

Department of Experimental Pharmacology, University of Naples 'Federico II', Via Domenico Montesano, 49-80131 Naples, Italy

[CA]Corresponding Author

**POSITIVELY charged macromolecules cause a variety of pathological events through their electrostatic interaction with anionic sites present on the membrane of target cells. In the present study we have investigated the effect of hyaluronic acid, a negatively charged molecule, on rat paw oedema induced by poly-L-lysine as well as on histamine release from rat mast cells and nitric oxide formation from rabbit aorta, both induced by this polycation. The results indicate that hyaluronic acid is able to suppress these poly-L-lysine induced effects with a mechanism which possibly depends on its negative charges which may balance the effects of positively charged polycations.**

**Key words:** Heparin, Histamine release, Hyaluronic acid, Nitric oxide, Oedema, Polycations

### Introduction

Positively charged macromolecules, such as cationic proteins and cationic polyamino acids, play important roles in a variety of biological systems. Poly-L-lysine and poly-L-arginine increase vascular permeability and cause oedema formation.[1] Polycations are able to release histamine from rat peritoneal cells.[2–4] Poly-L-lysine elicits the formation and release of nitric oxide (NO; for a review see Reference 5) from rat aortic rings and from bovine cultured aortic endothelial cells.[6,7]

These actions possibly depend on the electrostatic interaction of cationic macromolecules with anionic sites present on the membrane of target cells.[8,9] Thus heparin, a polyanionic molecule, is able to produce a marked inhibition of polycation induced oedema of the rat paw.[10] In addition, heparin inhibits histamine release from rat peritoneal mast cells stimulated by poly-L-lysine.[4]

Hyaluronic acid (HA), a negatively charged molecule, is a component of synovial fluid and plays an important role in the protection of the cartilage surface.[11] In this study we have investigated the effect of HA on rat paw oedema induced by poly-L-lysine as well as on histamine release from rat mast cells and NO formation from rabbit aorta, both induced by this polycation.

### Materials and Methods

*Materials:* Hyaluronic acid (mol. wt 670 000) was obtained from Fidia (Italy), poly-L-lysine (mol. wt 38 000) and all other reagents were from Sigma. Heparin had an activity of 166.9 U/mg.

*Paw oedema:* Oedema was induced in male Wistar rats (140–160 g) by subplantar injection into the left hind paw of 0.1 ml saline containing 1 mg poly-L-lysine. The volume of the paw was measured by plethysmometry (Basile, Italy) immediately after injection of poly-L-lysine as described previously.[12] Subsequent readings of the volume of the same paw were carried out at 30 or 60 min intervals and compared with the initial readings.

HA was injected into the paw together with poly-L-lysine (0.01–0.1–1 mg/paw) or subcutaneously (0.1–1–10 mg/kg) 1 h prior to the injection of the polyamino acid. In some experiments the effect of heparin (0.3 mg/paw) was also investigated.

*Histamine release:* Mixed peritoneal cells (approx. 5% mast cells) were obtained from male Wistar rats (200–300 g) according to Atkinson *et al.*[13]

Cells were washed and suspend in Tyrode's solution (pH 7.4). Aliquots of cell suspension ($4 \times 10^5$ to a final volume of 1 ml), were allowed to equilibrate at 37°C in a metabolic shaker with gentle agitation and histamine release was initiated by addition of poly-L-lysine (2.5 μg/ml) or concanavalin A (Con A, 5 μg/ml).

Experiments with Con A were carried out in the presence of phosphatidylserine (50 μg/ml). HA or heparin (1–10 μg/ml) were added to cell suspension 5 min before the inducer. The release was terminated after 10 min by addition of 2 ml ice-cold Tyrode's solution. Cells and supernatants were separated by centrifugation (150 × *g*, 10 min, 4°C) and histamine concentration in solutions and cells were quantified fluorimetrically.[14] Histamine release was expressed as a percentage of the total cellular histamine content.

All values were corrected for the spontaneous release occurring in the absence of the inducer (approx. 8%). Results are expressed as a percentage of the control release.

© 1994 Rapid Communications of Oxford Ltd

*Nitric oxide release:* Rabbit aortic rings (5–6 mm) were prepared from male albino rabbits (2–2.5 kg) and mounted in 10 ml organ chambers for recording tension. The rings were maintained at 37°C in Krebs' solution (pH 7.4). The solution was continuously gassed with 95% $O_2$ and 5% $CO_2$. Each ring was equilibrated under 2 g tension for 1 h in the presence of indomethacin $10^{-5}$ M. After equilibration, rings were contracted with phenylephrine (Phe, $10^{-7}$ M) and cumulative dose response curves ($1.25–20 \times 10^{-8}$ M) were obtained for the poly-L-lysine. HA (0.01–1 μg/ml) was added to the tissue bath 5 min prior to relaxing the ring with poly-L-lysine.

*Statistics:* Statistical analysis of the data was performed using a Pharm/PCS computer program. Means were compared by Student's *t*-test for unpaired data.

### Results

*Paw oedema:* The time course of poly-L-lysine oedema and its inhibition by HA (1 mg/paw) and heparin (0.3 mg/paw) are shown in Fig. 1. HA given locally at 0.01, 0.1 and 1 mg/paw exhibited a dose related inhibition of rat paw oedema by 10 ± 2%, 24 ± 3% ($p < 0.05$) and 35 ± 4% ($p < 0.05$), respectively (Fig. 2). In contrast HA, given systemically at doses up to 10 mg/kg did not inhibit poly-L-lysine induced oedema (data now shown).

*Histamine release:* Rat peritoneal cells stimulated with poly-L-lysine (2.5 μg/ml) released 46 ± 3% ($n = 6$) of the total cellular histamine content. The release induced by Con A (5 μg/ml) was 29 ± 1% ($n = 6$).

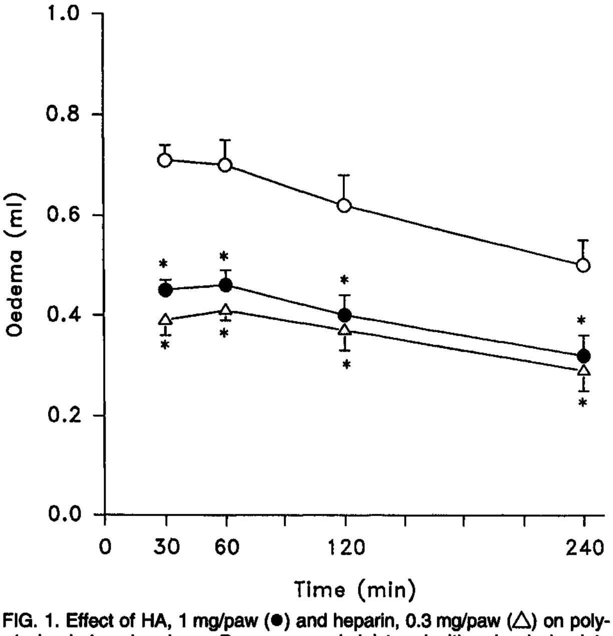

FIG. 1. Effect of HA, 1 mg/paw (●) and heparin, 0.3 mg/paw (△) on poly-L-lysine induced oedema. Drugs were administered with poly-L-lysine into the rat paw. The oedema induced by poly-L-lysine alone (control) is shown by open circles (O). Results are expressed as mean ± S.E.M. (vertical bars) of 5–6 rats; *$p < 0.05$ *vs.* control group.

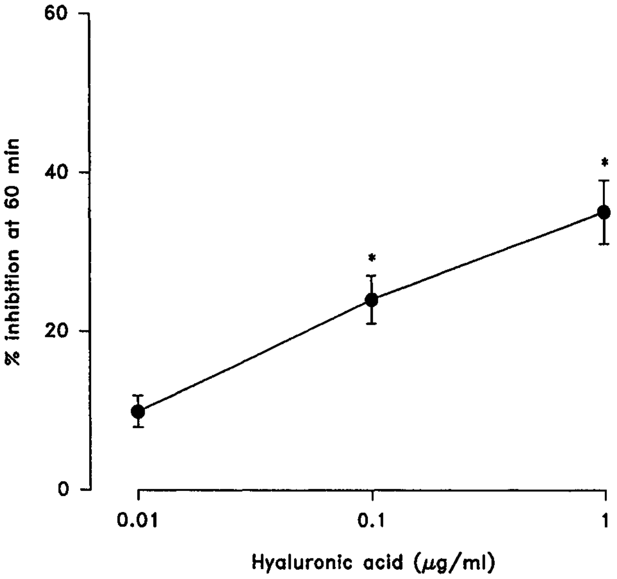

FIG. 2. Effect of HA on poly-L-lysine induced oedema. Results are expressed as percentage inhibition of the oedema occurring at 60 min in animals given poly-L-lysine alone. Each point is the mean ± S.E.M. (vertical bars) of 5–6 rats; *$p < 0.05$ *vs.* control group.

The effect of various concentrations of HA on the histamine release from these cells stimulated by poly-L-lysine or Con A is shown in Fig. 3. HA at 1 μg/ml moderately reduced (10 ± 6%) poly-L-lysine induced histamine release, while 3.3 and 10 μg/ml caused a significant ($p < 0.01$) inhibition of the release (44 ± 6% and 73 ± 7% respectively). In contrast HA at concentrations up to 10 μg/ml did not significantly modify the histamine release induced by Con A.

A similar profile of activity was shown by heparin. Thus the release of histamine was inhibited by 15 ± 4%, 89 ± 8% and 100% in presence of 1, 3.33 and

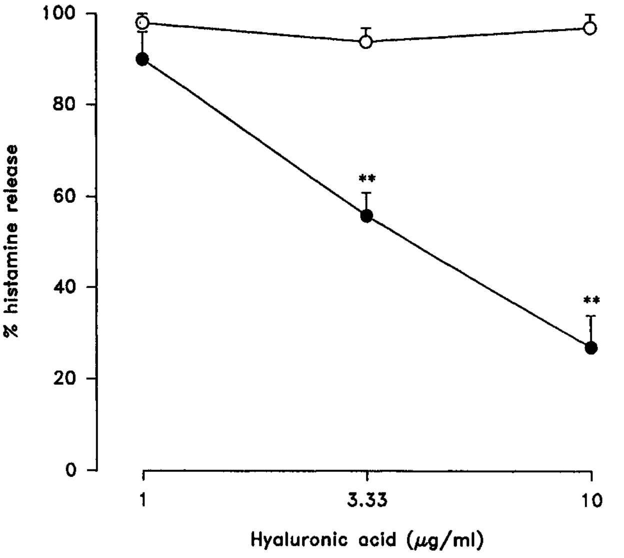

FIG. 3. Effect of HA on the histamine release from rat peritoneal cells induced by 2.5 μg/ml poly-L-lysine (●) or 5 μg/ml concanavalin A (O). Each point is the mean ± S.E.M. (vertical bars) of three duplicate experiments; **$p < 0.01$.

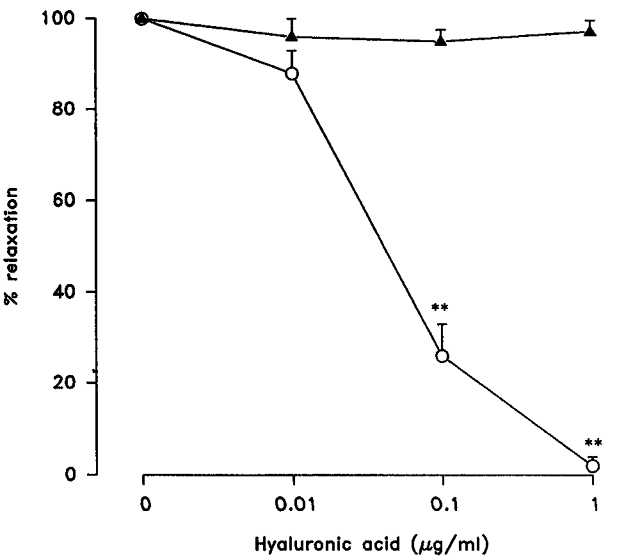

FIG. 4. Effect of HA on the relaxation of aortic rings pre-contracted with $1 \times 10^{-7}$ M phenylephrine induced by $5 \times 10^{-8}$ M poly-L-lysine (O) or $3 \times 10^{-7}$ M acetylcholine (▲). Experiments were carried out in the presence of $10^{-5}$ M indomethacin. $^{**}p < 0.01$.

10 μg/ml heparin, respectively, whereas the release induced by Con A was not affected (data not shown).

*Nitric oxide release:* Poly-L-lysine and acetylcholine produced a dose independent relaxation of aortic rings pre-contracted with $1 \times 10^{-7}$ M Phe.

Complete relaxation of (100%) of rings was induced by $5 \times 10^{-8}$ M poly-L-lysine or $3 \times 10^{-7}$ M acetylcholine. HA exhibited a concentration related inhibition of the relaxation induced by $5 \times 10^{-8}$ M poly-L-lysine while it was ineffective on the relaxation induced by $3 \times 10^{-7}$ M acetylcholine (Fig. 4).

### Discussion

The results show that HA is able to inhibit a variety of poly-L-lysine induced effects, such as oedema formation in the rat paw, histamine release from rat mast cells and NO release from rabbit aorta. It was found that the oedema induced by poly-L-lysine was characterized by rapid onset as shown in previous reports.[10]

Specific anionic sites have been visualized on capillary endothelium and it has been hypothesized that polycations increase vascular permeability and cause oedema formation by their electrostatic interaction with anionic sites.[9] The finding that HA, a negatively charged molecule, inhibits poly-L-lysine oedema only when given locally suggests that this compound is able to neutralize the positively charged groups of the polycations. In addition, it has been shown that polycations caused histamine release from rat peritoneal mast cells by interaction with anionic sites present on these cells.[4] Therefore, the finding that HA inhibits histamine release from rat mast cells induced by poly-L-lysine, but not that by Con A, is a further indication that HA may act through the neutralization of positive charges. On the other hand heparin, another negatively charged molecule, shows a similar profile of activity.[4]

Poly-L-lysine was found to induce the release of a relaxing factor from rat aorta and from bovine endothelial cells.[6,7] This factor has been identified as NO either because its release was abrogated by removing the endothelium[6] or it was suppressed by the NO synthase inhibitor $N^G$-nitro-L-arginine methyl ester.[7] HA inhibited the release of NO induced in rabbit aortic rings by poly-L-lysine, whereas it was unable to modify the NO release induced by acetylcholine. Endogenous NO is involved in inflammatory reactions since it has been shown that NO is formed at inflammatory sites and modulates oedema formation by increasing blood flow.[15]

In conclusion, taken together, the present results indicate that HA is able to suppress some pathological events induced by polycations, such as oedema formation and the release of some inflammatory mediators such as histamine and NO. The mechanism of action of HA possibly depends on its negative charges which may balance the effects of positively charged polycations. The relevance of these findings for the protective role of HA in the inflammatory cartilage damage deserves further investigation.

### References

1. Needham L, Hellewell PG, Williams TJ, Gordon JL. Endothelial functional responses and increased vascular permeability induced by polycations. *Lab Invest* 1988; **59:** 538–548.
2. Padawar J. The reaction of rat mast cells to polylysine. *J Cell Biol* 1970; **47:** 352–372.
3. Baxter JH, Adamik R. Differences in requirements and actions of various histamine releasing agents. *Biochem Pharmacol* 1978; **27:** 497–503.
4. Foreman JC, Lichtenstein LM. Induction of histamine secretion by polycations. *Biochem Biophys Acta* 1980; **629:** 587–603.
5. Moncada S, Palmer RMJ, Higgs EA. Nitric oxide: physiology, pathophysiology and pharmacology. *Pharmacol Rev* 1991; **43:** 109–142.
6. Thomas G, Hecker M, Ramwell PW. Vascular activity of polycations and basic amino acids: L-arginine does not specifically elicit endothelium-dependent relaxation. *Biochem Biophys Res Commun* 1989; **158:** 177–180.
7. Hecker M, Siegle I, Macarthur H, Sessa WC, Vane JR. Role of intracellular thiols in release of EDRF from cultured endothelial cells. *Am J Physiol* 1992; **262:** H888–H896.
8. Skutelsky E, Rudich Z, Danon D. Surface charge properties of the luminal front of blood vessel walls: an electron microscopical analysis. *Thromb Res* 1975; **7:** 623–634.
9. Skutelsky E, Danon D. Redistribution of surface anionic sites on the luminal front of blood vessel endothelium after interaction with polycationic ligand. *J Cell Biol* 1976; **71:** 232–241.
10. Antunes E, Mariano M, Cirino G, Levi S, de Nucci G. Pharmacological characterization of polycation-induced rat hind-paw oedema. *Br J Pharmacol* 1990; **101:** 986–990.
11. Sato H, Takahashi T, Ide H, *et al.* Antioxidant activity of synovial fluid, hyaluronic acid, and two subcomponents of hyaluronic acid. *Arthritis Rheum* 1988; **31:** 63–71.
12. Di Rosa M, Willoughby DA. Screens for anti-inflammatory drugs. *J Pharm Pharmacol* 1971; **23:** 297–298.
13. Atkinson G, Ennis M, Pearce FL. The effect of alkaline earth cations on the release of histamine from rat peritoneal mast cells treated with compound 48/80 and peptide 401. *Br J Pharmacol* 1979; **65:** 395–402.
14. Shore PA, Burkhalter A, Cohn VH. A method for the fluorimetric assay of histamine in tissues. *J Pharmacol Exp Ther* 1959; **127:** 182–186.
15. Ialenti A, Ianaro A, Moncada S, Di Rosa M. Modulation of acute inflammation by endogenous nitric oxide. *Eur J Pharmacol* 1992; **211:** 177–182.

**Received 7 February 1994;**
**accepted in revised form 12 April 1994**
